# SOCIAL ISOLATION AND DIET QUALITY AMONG US ADULTS WITH A DISABILITY IN NHANES, 2013–2018

**DOI:** 10.1093/geroni/igad104.2460

**Published:** 2023-12-21

**Authors:** Elizabeth Parker, Nadia Saif, Kathryn Barry, Jason Falvey, Odessa Addison

**Affiliations:** UMSOM, Baltimore, Maryland, United States; UMSOM, Baltimore, Maryland, United States; UMSOM, Baltimore, Maryland, United States; University of Maryland School of Medicine, Baltimore, Maryland, United States; University of Maryland School of Medicine, Baltimore, Maryland, United States

## Abstract

Social isolation and disability are established risk factors for poor nutrition. There is limited understanding of how social isolation affects diet quality specifically among adults with disability. This cross-sectional analysis used NHANES 2013-2018 data to assess whether social isolation is associated with diet quality among adults with disabilities. Adults with a disability, not pregnant/breastfeeding, and not missing dietary intake data were included (n=5,167). Disability was defined based on reporting difficulty with any activities of daily living, instrumental activities of daily living, lower extremity mobility activities, or general physical activities. Diet quality was assessed using the Healthy Eating Index (HEI); higher scores corresponded to higher diet quality. We computed a social isolation index by summing single status, living alone, and two social engagement difficulty measures (1 point for each component met; 4 points maximum). Multivariable linear regression, controlling for demographic and health covariates, estimated differences in HEI scores for dietary intake, by social isolation score. The highest social isolation score category was associated with lower total HEI score (β = -1.71 points, 95% CI -3.08, -0.34), and lower intake of vegetables (β = -0.39 points, 95% CI -0.59, -0.20) and seafood/plant proteins (β = -0.37 points, 95% CI -0.60, -0.13). Single status and both social engagement measures were associated with lower scores on certain adequacy components. There was little evidence of effect modification by age or gender (p-interaction >0.05). Among socially isolated adults with disabilities, increasing access to fresh foods, particularly vegetables and seafood/plant proteins, should be a priority.

